# Current practice of abdominal wall closure in elective surgery – Is there any consensus?

**DOI:** 10.1186/1471-2482-9-8

**Published:** 2009-05-15

**Authors:** Nuh N Rahbari, Phillip Knebel, Markus K Diener, Christoph Seidlmayer, Karsten Ridwelski, Hartmut Stöltzing, Christoph M Seiler

**Affiliations:** 1 Study Centre of the German Surgical Society, University of Heidelberg, Heidelberg, Germany; 2 Department of General and Visceral Surgery, St Bonifatius-Krankenhaus, Lingen, Germany; 3 Department of General and Visceral Surgery, Städtisches Klinikum, Magdeburg, Germany; 4 Department of General, Visceral, and Trauma Surgery, Robert-Bosch-Krankenhaus, Stuttgart, Germany

## Abstract

**Background:**

Development of incisional hernia after open abdominal surgery remains a major cause of post-operative morbidity. The aim of this study was to determine the current practice of surgeons in terms of access to and closure of the abdominal cavity in elective open surgery.

**Methods:**

Twelve surgical departments of the INSECT-Trial group documented the following variables for 50 consecutive patients undergoing abdominal surgery: fascial closure techniques, applied suture materials, application of subcutaneous sutures, subcutaneous drains, methods for skin closure. Descriptive analysis was performed and consensus of treatment variables was categorized into four levels: Strong consensus >95%, consensus 75–95%, overall agreement 50–75%, no consensus <50%.

**Results:**

157 out of 599 patients were eligible for analysis (85 (54%) midline, 54 (35%) transverse incisions). After midline incisions the fascia was closed continuously in 55 patients (65%), using slowly absorbable (n = 47, 55%), braided (n = 32, 38%) sutures with a strength of 1 (n = 48, 57%). In the transverse setting the fascia was closed continuously in 39 patients (72%) with slowly absorbable (n = 22, 41%) braided sutures (n = 27, 50%) with a strength of 1 (n = 30, 56%).

**Conclusion:**

In the present evaluation midline incision was the most frequently applied access in elective open abdominal surgery. None of the treatments for abdominal wall closure (except skin closure in the midline group) is performed on a consensus level.

## Background

Approximately 700.000 open abdominal procedures are performed annually in Germany and 4.000.000 in the United States [1]. Development of incisional hernia remains the major postoperative wound complication after open abdominal surgery with a stable incidence of 5% to 24% over the last decades [2,3]. Regarding pathogenesis of incisional hernias, the incision type (midline vs. transverse vs. oblique) and the strategy of fascia closure, i.e. the combination of applied suture technique (interrupted vs. continuous) and suture material (monofilament vs. braided; absorbable (rapidly, intermediately, slowly) vs. non-absorbable) are the main factors amenable to the surgeon.

There is still controversy about the best strategy for abdominal wall closure due to inconsistent and incomplete evidence provided by several randomized controlled trials (RCT) [4-8] as well as meta-analyses [9-12]. Based on the lack of data from well-designed long-term surgical trials, a large multi-centre randomized controlled trial (interrupted or continuous slowly absorbable sutures – evaluation of abdominal closure techniques, INSECT-Trial) recruited 624 patients between 2004 and 2006 with the rationale to compare the most relevant surgical practices for abdominal fascia closure after primary midline laparotomy [13]. Participating surgeons raised the following two questions during conduction of the INSECT-Trial:

1. Is midline incision still the most frequently applied access to the abdominal cavity in elective situations?

2. Has consensus for abdominal wall closure already been established in surgical routine?

The primary objective of this cross sectional study was therefore to identify current practice of abdominal cavity access and closure in elective surgery.

## Methods

### Study population

Twelve of the 25 participating centres of the INSECT-Trial group agreed in contribution to data acquisition (see acknowledgment). The local ethics committees of participating centres granted ethical approval for the study These include hospitals of all levels of health care (county/community, private, and university hospitals), thus ensuring representative study results. Surgeons at these hospitals were asked to assess surgical access and closure methods of 50 consecutive patients who underwent surgery at their service. Patients who underwent more than one operation during the study period were excluded.

### Study design

The study was designed as a cross-sectional cohort study and performed between July and August 2005. A standardized data entry form was designed in order to collect the following information: patient's gender and age, setting of surgery (elective vs. emergency surgery), surgical approach (open vs. laparoscopic surgery), history of previous abdominal surgery, type of applied abdominal incision (transverse vs. midline), suture material used for fascia closure (monofilament vs. braided, suture strength, absorption features), the technique used for fascia closure (interrupted sutures, continuous suture or combinations), the use of subcutaneous suture and subcutaneous drainage, and the method of skin closure. Classification of suture strengths was reported according to the United States Pharmacopeia XXII (1990).

All data entry forms were sent in an anonymous fashion to the Study Centre of the German Surgical Society and analyzed accordingly using Statistical Analysis System (SAS) version 9.1. Descriptive statistics were used in univariate analyses.

### Levels of consensus

The treatment of patients were categorized into four levels of consensus according to a classification of Hoffmann et al. [14]:

1. Strong consensus: treatment applied in >95% of cases

2. Consensus: treatment applied in 75–95% of cases

3. Overall agreement: treatment applied in 50–75% of cases

4. No consensus: treatment applied in <50% of cases

## Results

**Figure 1:**
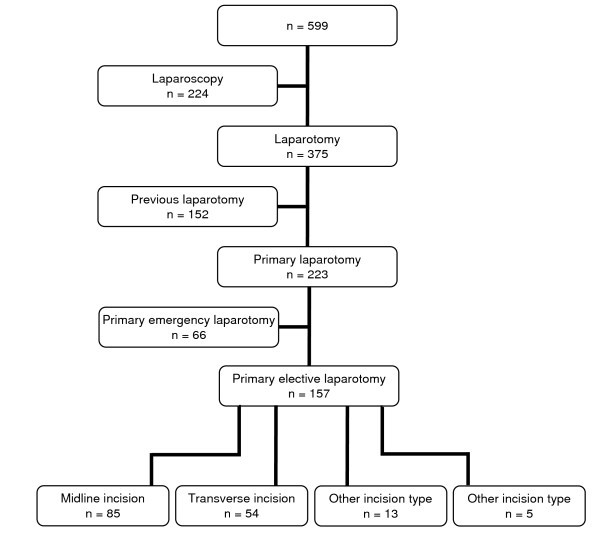
**Flow-chart on the number of available data entry forms and patients included in the final analysis**.

A total of 599 patient data entry forms out of 600 with an equal distribution of genders (49% men, 51% women) and a mean age of 57 years (SD 19 years) were available for analysis. An open approach was chosen in 375 patients (63%), a laparoscopic in 224 patients (37%). After exclusion of patients with previous open abdominal surgery, an emergency procedure, and laparoscopic procedures, a total of 157 patients with primary elective laparotomy were available for further analysis (Figure 1).

### Access to the abdominal cavity

Of all patients with elective primary laparotomy (n = 157), midline incisions were performed in 85 patients (54%), transverse incisions (crossing the midline) in 54 patients (35%) and miscellaneous incisions (e.g. paramedian and subcostal incisions) in 13 patients (8%).

### Closure of the abdominal wall

Fascia closure for midline incisions was performed with a continuous suture in 55 patients (65%) and interrupted sutures in 16 patients (19%), respectively. In 13 (15%) of these patients a combination of both techniques was applied. Monofilament sutures were applied for fascia closure in 51 patients (60%), braided sutures in 32 patients (38%). Non-absorbable sutures were chosen in four cases (5%), slowly absorbable sutures in 47 patients (55%), and moderately rapid absorbable sutures in 33 patients (39%). Sutures with the strength of 0 were used in two patients (2%), with strength of 1 in 48 patients (57%), and with strength of 2 in 31 patients (36%).

Subcutaneous suture was performed in 48 patients (57%) and a subcutaneous drain was placed in 19 patients (22%). Skin closure of patients who received midline incisions was performed with staples in 67 cases (79%).

In terms of consensus levels all technical features for closure of midline incisions met the level of "overall agreement", except the item of skin closure for which the level of "consensus" was reached (Table 1).

**Table 1:** Technical features for abdominal wall closure after elective and open primary midline laparotomies (n = 85)

**Parameter**	**Variables**	**Results**	**Level of consensus**
**Fascia suture technique**	Continuous	55 (65%)	Overall agreement
		
	Interrupted	16 (19%)	
		
	Combinations	13 (15%)	
		
	Missing	1 (1%)	

**Suture strength**	0	2 (2%)	Overall agreement
		
	1	48 (57%)	
		
	2	31 (36%)	
		
	Others	3 (4%)	
		
	Missing	1 (1%)	

**Suture material**	Monofilament	51 (60%)	Overall agreement
		
	Braided	32 (38%)	
		
	Missing	2 (2%)	

**Suture absorption**	No	4 (5%)	Overall agreement
		
	Slow	47 (55%)	
		
	Intermediate	33 (39%)	
		
	Rapid	0 (0%)	
		
	Missing	1 (1%)	

**Subcutaneous suture**	Yes	48 (57%)	Overall agreement
		
	No	36 (42%)	
		
	Missing	1 (1%)	

**Subcutaneous drainage**	Yes	19 (22%)	Overall agreement
		
	No	63 (74%)	
		
	Missing	3 (4%)	

**Skin closure**	Staples	67 (79%)	Consensus
		
	Suture	17 (20%)	
		
	Missing	1 (1%)	

Abdominal fascia of patients who had a transverse incision (n = 54) was sutured continuously in 39 patients (72%), interruptedly in eleven patients (20%), and with a combination of both techniques in two patients (4%). Suture material with the strength was 0 were chosen in 6 patients (11%), with a strength of 1 in 30 patients (56%) and with a strength of 2 in 17 patients (31%). Monofilament sutures were applied for fascia closure of 25 patients (46%) and braided sutures for 27 patients (50%). In two patients (4%) non-absorbable sutures, in 22 patients (41%) slowly absorbable sutures, in 29 patients (54%) moderately rapid absorbable sutures, and in one patient (2%) rapidly absorbable sutures were applied.

Thirty-nine patients (72%) received a subcutaneous suture; a subcutaneous drain was placed in 12 patients (22%). Skin closure of patients who received midline incisions was performed with staples in 39 cases (72%).

All technical features for closure of transverse incisions reached the level of "overall agreement" (Table 2).

**Table 2:** Technical features for abdominal wall closure after elective and open primary transverse laparotomies (n = 54)

**Parameter**	**Variables**	**Results**	**Level of Consensus**
**Fascia suture technique**	Continuous	39 (72%)	Overall agreement
		
	Interrupted	11 (20%)	
		
	Combinations	2 (4%)	
		
	Missing	2 (4%)	

**Suture strength**	0	6 (11%)	Overall agreement
		
	1	30 (56%)	
		
	2	17 (31%)	
		
	Others	1 (2%)	
		
	Missing	0 (0%)	

**Suture material**	Monofilament	25 (46%)	Overall agreement
		
	Braided	27 (50%)	
		
	Missing	2 (4%)	

**Suture absorption**	No	2 (4%)	Overall agreement
		
	Slow	22 (41%)	
		
	Intermediate	29 (54%)	
		
	Rapid	1 (2%)	
		
	Missing	0 (0%)	

**Subcutaneous suture**	Yes	39 (72%)	Overall agreement
		
	No	15 (28%)	
		
	Missing	0 (0%)	

**Subcutaneous drainage**	Yes	12 (22%)	Overall agreement
		
	No	40 (74%)	
		
	Missing	2 (4%)	

**Skin closure**	Staples	39 (72%)	Overall agreement
		
	Suture	15 (28%)	
		
	Missing	0 (0%)	

## Discussion

In the present analysis midline incision is still the most frequent access to the abdominal cavity in elective surgery. No consensus for fascia closure either for midline or transverse incisions in daily practice of abdominal surgery was detected (>75% of surgeons acting similarly). Use of staples for skin closure after midline incisions was the only item that reached the level of consensus.

Our finding that midline incisions are the most frequently applied access in open abdominal surgery is in accordance with previous reports [15]. While the abdominal access may to some degree depend on the target organ it was our aim to assess the overall frequency of midline incisions that would support the rationale of the INSECT-Trial. However, it appears valuable to perform further studies to evaluate the relative frequency of midline and transverse incisions in well-defined patient populations. The urgency of the intervention (i.e. elective vs. emergency laparotomy) as well as the target organ are critical factors to be considered for determination of the population under study. A recent Cochrane review indicated that transverse incisions are potentially less painful and less frequently associated with pulmonary complications, but failed to show a clear advantage for a reduction of incisional hernias [16] when compared to midline incisions. Further data analyzing the patient's perspective (i.e. post-operative pain) in a blinded randomized fashion are needed for evidence-based surgical decision making. Currently, both incision types may be used in daily practice of elective surgery depending on the surgeons preference.

The lack of consensus for abdominal wall closure strategies after midline incisions demonstrates persistent uncertainty within the surgical community. Several RCTs [4-8] and meta-analyses [9-12] were published comparing different closure methods of midline abdominal incisions. In a critical appraisal of meta-analyses in the surgical literature [17] two of these meta-analyses have been found to be of low methodological quality [9,11]. The meta-analysis by Hodgson et al. was of high quality and reported significantly less incisional hernias after closure with continuous non-absorbable sutures but also found significantly more suture sinuses and wound pain requiring further interventions. This material was not used in the present survey which demonstrates the harm caused by this material in practice. Unfortunately, the authors compared absorbable and non-absorbable materials only and did not distinguish between rapidly and slowly absorbing sutures [10,12]. Therefore, a further meta-analysis was performed by van't Riet et al. [12] which was not evaluated in the above mentioned critical appraisal, but can be considered to be well-designed according to the methods of the Cochrane collaboration. However, it failed to show a clear superiority of one strategy regarding the prevention of incisional hernia, if all details of closure technique, closure material, and needle type are taken into account [12]. In particular, it could not demonstrate a significant advantage between interrupted rapidly absorbable and continuous slowly absorbable sutures in terms of incisional hernia development. The rationale of the INSECT-Trial was based on these findings [13].

According to our data, there is no consensus in closure of transverse incisions either. Closure technique of the abdominal fascia with a continuous suture and skin closure with staples were the only items that almost reached the level of consensus (both performed in 72%). In contrast to midline incisions, however, so far no randomized trial has been solely conducted in order to investigate closure techniques in this setting. Hence, the ideal closure strategy is still to be determined. From some trials comparing closure techniques in midline incisions [5,18,19] part of the data, concerning transverse closure procedures only, can be extracted. Looking at these subgroups, results show approximately equal incidence of incisional hernias between 4% and 10% in interrupted versus continuous closure procedures. However, these trials have not been designed to prove equivalence or superiority of either technique. Data from further high-quality RCTs are awaited in order to resolve this open question.

The results of our study might be affected due to bias. The participating hospitals of the INSECT-Trial who agreed to contribute to this study may be a subgroup of hospitals dedicated to clinical research and therefore act differently to other hospitals (e.g. surgeons agree to randomize their patients). However, it is unlikely that such an effect might have systematically shifted the results in favour of consensus.

A cross-sectional consecutive data collection of real patient data seems to be the optimal design to answer our initial study questions. Selection bias within the hospitals is prevented due to a consecutive recruitment and measurement bias due to standardized data collection. Furthermore, surgeons of the INSECT-Trial group were trained in data collection during specific preparatory INSECT-Trial meetings [20]. Other study designs (e.g. surveys) have been used and detected, similar to our findings, considerable differences between available evidence and daily practice in abdominal wall closure [21]. However, large, prospective cohort studies would be required to more ultimatel answer the question what the most frequently applied abdominal access and fascial closure techniques are. These studies would require separate study management and in particular extra funding. We are therefore confindent that our data may still provide a valuable picture of current practice.

Currently, no clinical guidelines are available for abdominal wall closure after any type of incision. Further well designed pragmatic randomized controlled trials are necessary to support a development of such evidence-based guidelines and to change the current practice. Otherwise a reduction of incisional hernias seems unfeasible and treatment remains on the level of "overall agreement". The results of the recently published INSECT-Trial certainly contribute to the body of evidence on abdominal fascia closure techniques[22]. Furthermore, the unexpected high incidence of incisional hernias in all study arms warrants further research in this field.

## Conclusion

In conclusion, midline incisions were the predominant type of abdominal access in the present study of participating centers of the INSECT-Trial. There is no consensus regarding the strategy for closure of the abdominal fascia after midline and transverse incisions.

## Competing interests

The authors declare that they have no competing interests.

## Authors' contributions

NNR: Study design, data analysis and interpretation, drafting of the manuscript PK: Study design, data acquisition, data analysis, revsion of the manuscript. MKD: Data acquisition, analysis and interpretation, revsion of the manuscript. CS: Study design, data acquisition, data interpretation, revision of the manuscript. KR: Study design, data acquisition, data interpretation, revision of the manuscript. HS: Study design, data acquisition, data interpretation, revision of the manuscript. CMS: Study design, data acquisition, data analysis and interpretation, drafting of the manuscript.

## Pre-publication history

The pre-publication history for this paper can be accessed here:


